# *N*-(4-Chloro­phen­yl)quinolin-2-amine

**DOI:** 10.1107/S1600536811045521

**Published:** 2011-11-05

**Authors:** Zainal Abidin Hasan, Zanariah Abdullah, Hairul Anuar Tajuddin, Seik Weng Ng, Edward R. T. Tiekink

**Affiliations:** a Department of Chemistry, University of Malaya, 50603 Kuala Lumpur, Malaysia; b Chemistry Department, Faculty of Science, King Abdulaziz University, PO Box 80203 Jeddah, Saudi Arabia

## Abstract

There is a twist in the title mol­ecule, C_15_H_11_ClN_2_, as seen in the dihedral angle of 18.85 (9)° between the quinoline and benzene rings. A short C—H⋯N contact arises from this conformation and the amine H and quinoline N atoms are directed towards opposite sides of the mol­ecule. In the crystal, supra­molecular layers in the *ab* plane are mediated by C—H⋯π inter­actions.

## Related literature

For the structure of a related pyridine amine derivative, see: Aznan Akhmad *et al.* (2010 ▶).
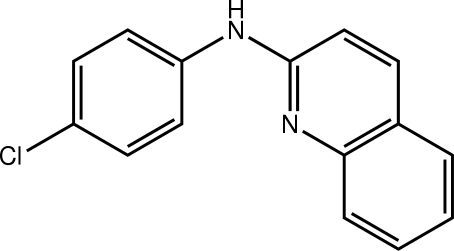


## Experimental

### 

#### Crystal data

C_15_H_11_ClN_2_
*M* *_r_* = 254.71
Monoclinic, 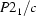

*a* = 5.9565 (4) Å
*b* = 7.9936 (6) Å
*c* = 25.0603 (18) Å
β = 92.744 (1)°
*V* = 1191.85 (15) Å^3^
*Z* = 4
Mo *K*α radiation
μ = 0.30 mm^−1^
*T* = 100 K
0.2 × 0.1 × 0.1 mm

#### Data collection

Bruker SMART APEX CCD diffractometer
Absorption correction: multi-scan (*SADABS*; Sheldrick, 1996 ▶) *T*_min_ = 0.872, *T*_max_ = 1
10754 measured reflections
2726 independent reflections
2460 reflections with *I* > 2σ(*I*)
*R*_int_ = 0.030

#### Refinement

*R*[*F* ^2^ > 2σ(*F* ^2^)] = 0.052
*wR*(*F* ^2^) = 0.134
*S* = 1.11
2726 reflections
167 parameters
1 restraint
H atoms treated by a mixture of independent and constrained refinement
Δρ_max_ = 0.50 e Å^−3^
Δρ_min_ = −0.32 e Å^−3^

### 

Data collection: *APEX2* (Bruker, 2009 ▶); cell refinement: *SAINT* (Bruker, 2009 ▶); data reduction: *SAINT*; program(s) used to solve structure: *SHELXS97* (Sheldrick, 2008 ▶); program(s) used to refine structure: *SHELXL97* (Sheldrick, 2008 ▶); molecular graphics: *ORTEP-3* (Farrugia, 1997 ▶) and *DIAMOND* (Brandenburg, 2006 ▶); software used to prepare material for publication: *publCIF* (Westrip, 2010 ▶).

## Supplementary Material

Crystal structure: contains datablock(s) global, I. DOI: 10.1107/S1600536811045521/hb6472sup1.cif

Structure factors: contains datablock(s) I. DOI: 10.1107/S1600536811045521/hb6472Isup2.hkl

Supplementary material file. DOI: 10.1107/S1600536811045521/hb6472Isup3.cml

Additional supplementary materials:  crystallographic information; 3D view; checkCIF report

## Figures and Tables

**Table 1:** Hydrogen-bond geometry (Å, °) *Cg*1 *Cg*2 and *Cg*3 are the centroids of of the N1,C7–C10,C15, C10–C15 and C1–C6 rings, respectively.

*D*—H⋯*A*	*D*—H	H⋯*A*	*D*⋯*A*	*D*—H⋯*A*
C2—H2⋯N2	0.95	2.39	2.961 (3)	118
C3—H3⋯*Cg*1^i^	0.95	2.94	3.734 (3)	142
C9—H9⋯*Cg*2^ii^	0.95	2.79	3.383 (3)	121
C14—H14⋯*Cg*2^i^	0.95	2.83	3.440 (3)	123
C6—H6⋯*Cg*3^ii^	0.95	2.81	3.590 (3)	140

Symmetry codes: (i) 
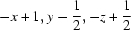
; (ii) 
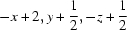
.

**Fig. 1.**
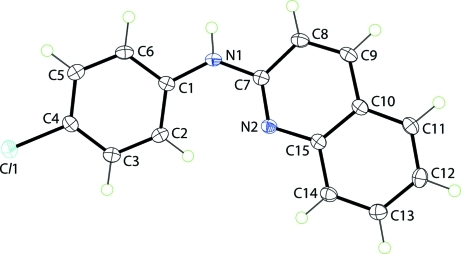
The molecular structure of (I) showing displacement ellipsoids at the 50% probability level.

**Fig. 2.**
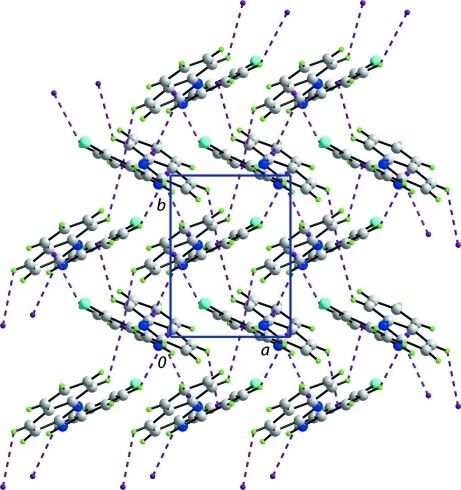
Layers in the ab plane in (I) sustained by C—H···π interactions shown as orange dashed lines.

**Fig. 3.**
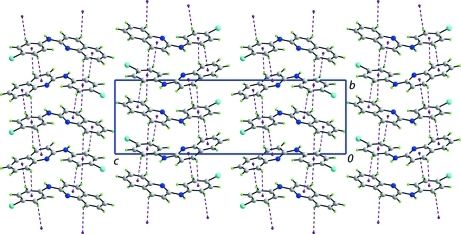
Unit-cell contents for (I) shown in projection down the a axis highlighting the stacking of layers. The C—H···π interactions are shown as orange dashed lines.

## supplementary crystallographic information

### Comment

As a part of on-going studies of nitropyridine derivatives (Aznan Akhmad *et al.*, 2010), the title compound was synthesized and structurally characterized. In (I), Fig. 1, the dihedral angle between the quinolinyl [r.m.s. deviation for the 10 non-hydrogen atoms = 0.022 Å] and benzene rings is 18.85 (9) Å indicating a twist in the molecule. The amine-H and quinolinyl-N atoms are directed towards opposite sides of the molecule. The quinolinyl-N atom participates in a close intramolecular C—H···N contact, Table 1. The amine-H atom is flanked by aromatic residues precluding its participation in close intermolecular contacts. The molecules are connected into supramolecular layers in the *ab* plane *via* C—H···π interactions, Fig. 2 and Table 1. Layers are connected along the *c* axis *via* weak C—H···Cl contacts, with the shortest of these being a C12—H12···Cl1^i^ contact of 2.92 Å [C12···Cl1^i^ = 3.655 (3) Å with angle at H12 = 135 °, for *i*: 1 + *x*, 1/2 - *y*, -1/2 + *z*], Fig. 3.

### Experimental

2-Chloroquinoline (1.0 g, 0.006 mol) was added to a solution of 4-chloroaniline (0.78 g, 0.006 mol) in ethanol (10 ml), and the mixture was refluxed for 7 h. The mixture was then cooled and the solvent evaporated off. The residue was dissolved in water and then extracted with diethyl ether (3 *x* 10 ml). The ether extracts were washed with water (3 *x* 10 ml) and dried over anhydrous sodium sulfate. Evaporation of the solvent gave the product and crystallization from its ethanol solution gave colourless prisms.

### Refinement

Carbon-bound hydrogen atoms were placed at calculated positions (C—H 0.95 Å) and were treated as riding on their parent carbon atoms, with *U*(H) set to 1.2 times *U*_eq_(C). The amine-H atom was refined with N—H = 0.86±0.01 Å with refined *U*_iso_.

### Crystal data

**Table:**

C_15_H_11_ClN_2_	*F*(000) = 528
*M**_r_* = 254.71	*D*_x_ = 1.419 Mg m^−^^3^
Monoclinic, *P*2_1_/*c*	Mo *K*α radiation, λ = 0.71073 Å
Hall symbol: -P 2ybc	Cell parameters from 5281 reflections
*a* = 5.9565 (4) Å	θ = 2.7–28.8°
*b* = 7.9936 (6) Å	µ = 0.30 mm^−^^1^
*c* = 25.0603 (18) Å	*T* = 100 K
β = 92.744 (1)°	Prism, colourless
*V* = 1191.85 (15) Å^3^	0.2 × 0.1 × 0.1 mm
*Z* = 4	

### Data collection

**Table:**

Bruker SMART APEX CCD diffractometer	2726 independent reflections
Radiation source: fine-focus sealed tube	2460 reflections with *I* > 2σ(*I*)
graphite	*R*_int_ = 0.030
ω scans	θ_max_ = 27.5°, θ_min_ = 1.6°
Absorption correction: multi-scan (*SADABS*; Sheldrick, 1996)	*h* = −7→7
*T*_min_ = 0.872, *T*_max_ = 1	*k* = −9→10
10754 measured reflections	*l* = −32→31

### Refinement

**Table:**

Refinement on *F*^2^	Primary atom site location: structure-invariant direct methods
Least-squares matrix: full	Secondary atom site location: difference Fourier map
*R*[*F*^2^ > 2σ(*F*^2^)] = 0.052	Hydrogen site location: inferred from neighbouring sites
*wR*(*F*^2^) = 0.134	H atoms treated by a mixture of independent and constrained refinement
*S* = 1.11	*w* = 1/[σ^2^(*F*_o_^2^) + (0.0396*P*)^2^ + 2.5503*P*] where *P* = (*F*_o_^2^ + 2*F*_c_^2^)/3
2726 reflections	(Δ/σ)_max_ = 0.001
167 parameters	Δρ_max_ = 0.50 e Å^−^^3^
1 restraint	Δρ_min_ = −0.32 e Å^−^^3^

### Special details

**Table:**

Refinement. Refinement of *F*^2^ against ALL reflections. The weighted *R*-factor *wR* and goodness of fit *S* are based on *F*^2^, conventional *R*-factors *R* are based on *F*, with *F* set to zero for negative *F*^2^. The threshold expression of *F*^2^ > 2σ(*F*^2^) is used only for calculating *R*-factors(gt) *etc*. and is not relevant to the choice of reflections for refinement. *R*-factors based on *F*^2^ are statistically about twice as large as those based on *F*, and *R*- factors based on ALL data will be even larger.

### Fractional atomic coordinates and isotropic or equivalent isotropic displacement parameters (Å^2^)

**Table:**

	*x*	*y*	*z*	*U*_iso_*/*U*_eq_	
Cl1	0.28861 (10)	0.29464 (9)	0.44653 (2)	0.02527 (18)	
N1	0.8994 (3)	0.5543 (3)	0.28636 (8)	0.0188 (4)	
H1n	1.015 (4)	0.601 (4)	0.3019 (11)	0.032 (9)*	
N2	0.7753 (3)	0.4313 (2)	0.20524 (8)	0.0162 (4)	
C1	0.7450 (4)	0.4911 (3)	0.32189 (9)	0.0155 (4)	
C2	0.5367 (4)	0.4212 (3)	0.30617 (9)	0.0170 (5)	
H2	0.4911	0.4148	0.2694	0.020*	
C3	0.3967 (4)	0.3611 (3)	0.34480 (9)	0.0174 (5)	
H3	0.2554	0.3131	0.3344	0.021*	
C4	0.4632 (4)	0.3714 (3)	0.39834 (9)	0.0173 (5)	
C5	0.6687 (4)	0.4413 (3)	0.41464 (9)	0.0177 (5)	
H5	0.7132	0.4479	0.4515	0.021*	
C6	0.8072 (4)	0.5010 (3)	0.37631 (9)	0.0175 (5)	
H6	0.9475	0.5498	0.3871	0.021*	
C7	0.9269 (4)	0.5194 (3)	0.23314 (9)	0.0161 (5)	
C8	1.1286 (4)	0.5837 (3)	0.21166 (10)	0.0187 (5)	
H8	1.2304	0.6492	0.2332	0.022*	
C9	1.1715 (4)	0.5496 (3)	0.16038 (10)	0.0187 (5)	
H9	1.3059	0.5891	0.1458	0.022*	
C10	1.0152 (4)	0.4543 (3)	0.12822 (9)	0.0165 (5)	
C11	1.0462 (4)	0.4160 (3)	0.07422 (9)	0.0192 (5)	
H11	1.1799	0.4505	0.0582	0.023*	
C12	0.8849 (4)	0.3294 (3)	0.04451 (9)	0.0202 (5)	
H12	0.9065	0.3045	0.0080	0.024*	
C13	0.6878 (4)	0.2778 (3)	0.06850 (10)	0.0195 (5)	
H13	0.5754	0.2194	0.0478	0.023*	
C14	0.6552 (4)	0.3104 (3)	0.12125 (9)	0.0181 (5)	
H14	0.5225	0.2718	0.1368	0.022*	
C15	0.8163 (4)	0.4006 (3)	0.15264 (9)	0.0151 (4)	

### Atomic displacement parameters (Å^2^)

**Table:**

	*U*^11^	*U*^22^	*U*^33^	*U*^12^	*U*^13^	*U*^23^
Cl1	0.0228 (3)	0.0326 (4)	0.0205 (3)	−0.0052 (3)	0.0018 (2)	0.0039 (2)
N1	0.0163 (10)	0.0204 (11)	0.0195 (10)	−0.0052 (8)	−0.0010 (8)	−0.0027 (8)
N2	0.0159 (9)	0.0145 (9)	0.0181 (9)	−0.0013 (7)	−0.0003 (7)	0.0009 (7)
C1	0.0143 (10)	0.0114 (10)	0.0207 (11)	0.0013 (8)	0.0012 (8)	−0.0004 (8)
C2	0.0172 (11)	0.0155 (11)	0.0179 (11)	0.0027 (9)	−0.0025 (8)	−0.0019 (9)
C3	0.0146 (10)	0.0155 (11)	0.0221 (11)	0.0017 (9)	−0.0001 (8)	−0.0009 (9)
C4	0.0170 (11)	0.0146 (11)	0.0204 (11)	0.0007 (9)	0.0036 (9)	0.0008 (9)
C5	0.0185 (11)	0.0157 (11)	0.0187 (11)	0.0035 (9)	−0.0017 (8)	−0.0023 (9)
C6	0.0153 (10)	0.0146 (11)	0.0223 (11)	0.0002 (8)	−0.0023 (8)	−0.0032 (9)
C7	0.0155 (10)	0.0138 (11)	0.0189 (11)	0.0017 (9)	−0.0008 (8)	0.0023 (9)
C8	0.0171 (11)	0.0155 (11)	0.0232 (12)	−0.0031 (9)	−0.0025 (9)	0.0017 (9)
C9	0.0147 (10)	0.0164 (11)	0.0250 (12)	−0.0023 (9)	0.0012 (9)	0.0037 (9)
C10	0.0162 (11)	0.0129 (11)	0.0204 (11)	0.0004 (9)	0.0011 (8)	0.0035 (9)
C11	0.0176 (11)	0.0179 (12)	0.0223 (12)	0.0000 (9)	0.0039 (9)	0.0045 (9)
C12	0.0256 (12)	0.0183 (12)	0.0169 (11)	0.0035 (10)	0.0027 (9)	0.0027 (9)
C13	0.0200 (11)	0.0165 (11)	0.0215 (11)	−0.0009 (9)	−0.0019 (9)	0.0019 (9)
C14	0.0180 (11)	0.0150 (11)	0.0211 (11)	−0.0023 (9)	0.0000 (9)	0.0023 (9)
C15	0.0156 (10)	0.0117 (10)	0.0179 (11)	0.0018 (8)	0.0002 (8)	0.0040 (8)

### Geometric parameters (Å, °)

**Table:**

Cl1—C4	1.742 (2)	C7—C8	1.435 (3)
N1—C7	1.380 (3)	C8—C9	1.350 (3)
N1—C1	1.404 (3)	C8—H8	0.9500
N1—H1n	0.858 (10)	C9—C10	1.423 (3)
N2—C7	1.319 (3)	C9—H9	0.9500
N2—C15	1.374 (3)	C10—C11	1.408 (3)
C1—C2	1.400 (3)	C10—C15	1.425 (3)
C1—C6	1.398 (3)	C11—C12	1.374 (3)
C2—C3	1.393 (3)	C11—H11	0.9500
C2—H2	0.9500	C12—C13	1.406 (3)
C3—C4	1.383 (3)	C12—H12	0.9500
C3—H3	0.9500	C13—C14	1.370 (3)
C4—C5	1.389 (3)	C13—H13	0.9500
C5—C6	1.381 (3)	C14—C15	1.410 (3)
C5—H5	0.9500	C14—H14	0.9500
C6—H6	0.9500		
			
C7—N1—C1	130.7 (2)	C9—C8—C7	119.0 (2)
C7—N1—H1n	113 (2)	C9—C8—H8	120.5
C1—N1—H1n	114 (2)	C7—C8—H8	120.5
C7—N2—C15	117.1 (2)	C8—C9—C10	119.9 (2)
C2—C1—C6	119.1 (2)	C8—C9—H9	120.0
C2—C1—N1	124.3 (2)	C10—C9—H9	120.0
C6—C1—N1	116.6 (2)	C11—C10—C9	123.3 (2)
C3—C2—C1	119.6 (2)	C11—C10—C15	119.8 (2)
C3—C2—H2	120.2	C9—C10—C15	116.9 (2)
C1—C2—H2	120.2	C12—C11—C10	120.7 (2)
C4—C3—C2	120.0 (2)	C12—C11—H11	119.7
C4—C3—H3	120.0	C10—C11—H11	119.7
C2—C3—H3	120.0	C11—C12—C13	119.5 (2)
C5—C4—C3	121.1 (2)	C11—C12—H12	120.3
C5—C4—Cl1	119.00 (18)	C13—C12—H12	120.3
C3—C4—Cl1	119.92 (18)	C14—C13—C12	121.1 (2)
C6—C5—C4	118.8 (2)	C14—C13—H13	119.5
C6—C5—H5	120.6	C12—C13—H13	119.5
C4—C5—H5	120.6	C13—C14—C15	120.8 (2)
C5—C6—C1	121.3 (2)	C13—C14—H14	119.6
C5—C6—H6	119.3	C15—C14—H14	119.6
C1—C6—H6	119.3	N2—C15—C14	118.6 (2)
N2—C7—N1	120.7 (2)	N2—C15—C10	123.2 (2)
N2—C7—C8	123.8 (2)	C14—C15—C10	118.2 (2)
N1—C7—C8	115.5 (2)		
			
C7—N1—C1—C2	−23.3 (4)	N1—C7—C8—C9	177.6 (2)
C7—N1—C1—C6	157.0 (2)	C7—C8—C9—C10	1.3 (4)
C6—C1—C2—C3	−0.8 (3)	C8—C9—C10—C11	178.9 (2)
N1—C1—C2—C3	179.6 (2)	C8—C9—C10—C15	0.6 (3)
C1—C2—C3—C4	0.3 (3)	C9—C10—C11—C12	−177.3 (2)
C2—C3—C4—C5	0.1 (4)	C15—C10—C11—C12	0.9 (3)
C2—C3—C4—Cl1	−179.50 (18)	C10—C11—C12—C13	−0.4 (4)
C3—C4—C5—C6	0.0 (4)	C11—C12—C13—C14	−0.9 (4)
Cl1—C4—C5—C6	179.60 (18)	C12—C13—C14—C15	1.7 (4)
C4—C5—C6—C1	−0.5 (3)	C7—N2—C15—C14	−178.4 (2)
C2—C1—C6—C5	0.9 (3)	C7—N2—C15—C10	2.2 (3)
N1—C1—C6—C5	−179.4 (2)	C13—C14—C15—N2	179.5 (2)
C15—N2—C7—N1	−179.4 (2)	C13—C14—C15—C10	−1.0 (3)
C15—N2—C7—C8	0.0 (3)	C11—C10—C15—N2	179.2 (2)
C1—N1—C7—N2	10.6 (4)	C9—C10—C15—N2	−2.5 (3)
C1—N1—C7—C8	−168.8 (2)	C11—C10—C15—C14	−0.2 (3)
N2—C7—C8—C9	−1.8 (4)	C9—C10—C15—C14	178.1 (2)

### Hydrogen-bond geometry (Å, °)

**Table:**

Cg1 Cg2 and Cg3 are the centroids of of the N1,C7–C10,C15, C10–C15 and C1–C6 rings, respectively.

**Table:**

*D*—H···*A*	*D*—H	H···*A*	*D*···*A*	*D*—H···*A*
C2—H2···N2	0.95	2.39	2.961 (3)	118
C3—H3···Cg1^i^	0.95	2.94	3.734 (3)	142
C9—H9···Cg2^ii^	0.95	2.79	3.383 (3)	121
C14—H14···Cg2^i^	0.95	2.83	3.440 (3)	123
C6—H6···Cg3^ii^	0.95	2.81	3.590 (3)	140

Symmetry codes: (i) −*x*+1, *y*−1/2, −*z*+1/2; (ii) −*x*+2, *y*+1/2, −*z*+1/2.
